# Association between visual impairment and sleep duration: analysis of the 2009 National Health Interview Survey (NHIS)

**DOI:** 10.1186/1471-2415-14-115

**Published:** 2014-10-01

**Authors:** Alberto R Ramos, Douglas M Wallace, Natasha J Williams, David Warren Spence, Seithikurippu Ratnas Pandi-Perumal, Ferdinand Zizi, Girardin Jean-Louis

**Affiliations:** Department of Neurology, Miller School of Medicine, University of Miami, Miami, FL USA; Center for Healthful Behavior Change (CHBC), Division of Health and Behavior, Department of Population Health, NYU Langone Medical Center, 227 East 30th Street, Floor # 6-615, New York, NY 10016 USA; 652 Dufferin Street, Toronto, ON M6K 2B4 Canada

**Keywords:** Visual Impairment, Sleep Duration, Race/ethnicity

## Abstract

**Background:**

Visual impairment (VI) is associated with increased mortality and health factors such as depression and cardiovascular disease. Epidemiologic studies consistently show associations between sleep duration with adverse health outcomes, but these have not systematically considered the influence of VI. The aim of this study was to ascertain the independent association between VI and sleep duration using the National Health Interview Survey (NHIS) data. We also examined whether race/ethnicity influenced these associations independently of sociodemographic and medical characteristics.

**Methods:**

Our analysis was based on the 2009 NHIS, providing valid sleep and vision data for 29,815 participants. The NHIS is a cross-sectional household interview survey utilizing a multistage area probability design. Trained personnel from the US census bureau gathered data during face-to-face interview and obtained socio-demographic, self-reported habitual sleep duration and physician-diagnosed chronic conditions.

**Results:**

The mean age of the sample was 48 years and 56% were female. Short sleep and long sleep durations were reported by 49% and 23% of the participants, respectively. Visual impairment was observed in 10%. Multivariate-adjusted logistic regression models showed significant associations between VI and short sleep (OR = 1.6, 95% CI = 1.5-1.9 and long sleep durations (OR = 1.6, 95% CI = 1.3-1.9). These associations persisted in multivariate models stratified by race-ethnic groups.

**Conclusion:**

Visual impairment was associated with both short and long sleep durations. Analysis of epidemiologic sleep data should consider visual impairment as an important factor likely to influence the amount of sleep experienced habitually.

## Background

Visual impairment (VI) has been associated with increased mortality and adverse health factors such as depression and cardiovascular disease [1–3]. Few studies have observed that visually impaired individuals experience increased frequency of sleep symptoms and sleep/wake disorders [4–7]. Sleep difficulties, documented from clinical samples, include fragmented sleep, increased sleep onset latency, short sleep duration, and daytime naps [7]. Similar findings have been observed in population based studies from U.S. [6] and Swedish volunteers [8].

Epidemiological studies have linked sleep duration with increased mortality and cardio-metabolic disease [9]. Short sleep (<5-6 hours) and long sleep durations (>8-9 hours) are associated to obesity, heart disease, stroke, diabetes mellitus, hypertension, and mood disturbances [9–11], but visual impairment have not been considered as an important factor in these studies. Within the purview of public health challenges associated with inadequate sleep, it has become important to assess the risk of medical comorbidities, such as visual impairment, that may be associated with sleeping below or above the modal population sleep time.

In light of the epidemiologic and clinical evidence that independently links adverse health outcomes with sleep duration and visual impairment; we sought to examine the associations between VI with sleep duration. Studies on the association between visual impairment and sleep [4–8] have been conducted in clinical samples or in elderly population-based studies, which limit their external validity. In contrast, this analysis was conducted among a representative sample of the US population with a diverse age, sex and ethnic background. Relevant to this point is evidence that race-ethnic disparities exist in disorders that may cause visual impairment (i.e. diabetic retinopathy, glaucoma) [12, 13], sleep duration [14] and cardio-metabolic risk, [15, 16] such that individuals of the black race-ethnicity have a greater burden of visual impairment as well as an increased prevalence of both short and long sleep when compared with white counterparts [17, 18].

The aim of this study was to evaluate the associations between visual impairment and self-reported sleep duration in a representative sample of the US adult population, using data from the 2009 National Health Interview Survey (NHIS). Our hypothesis was that visual impairment is independently associated with short and long sleep durations. We also examined the associations among race/ethnicity, sleep duration and visual impairment, and whether these factors were independent of individual’s sociodemographic and medical characteristics.

## Methods

The study sample was derived from the 2009 NHIS data. The NHIS is conducted annually by the National Center for Health Statistics (NCHS), Centers for Disease Control and Prevention (CDC). The procedures involved in the NHIS and details concerning its sample design can be found in "NHIS Survey Description – 2009 Public Use Data Release" [19]. Briefly, the NHIS survey comprises a complex multistage area probability design that provides a representative sample of US households from all 50 states and the District of Columbia. In 2009, the interviewed sample consisted of 33,856 households, which yielded 88,446 persons in 34,640 families. There was a 65.4% response rate from the 34,616 eligible adults obtained from the Sample Adult questionnaire. Face-to-face interviews were conducted by trained interviewers from the US Census Bureau. The surveys were conducted using computer-assisted personal interviewing. Information was collected on socio-demographic data (age, sex, race/ethnicity, average family income, and education), health risks (smoking status and alcohol intake), and physician diagnosed chronic conditions or diseases such as hypertension, diabetes, coronary heart disease, cancer, kidney disease, stroke, and myocardial infarction.

The NHIS is a public data repository that is freely accessible online and can be downloaded and coded by an experienced bio-statistician using available coding and analytic instructions provided by the CDC. The research was conducted in accordance with the Declaration of Helsinki and was approved by the ethics committee [19].

### Visual impairment

Participants reported visual impairment by answering a series of questions, as initiated by the Vision Health Initiative (VHI) branch of the CDC [20, 21]. The interview documented whether a physician or other health professional ever informed participants that they had diabetic retinopathy, cataracts, glaucoma, or macular degeneration. Participants also reported if they were blind or unable to see, had trouble seeing even with corrective glasses or contacts, had a condition or health problem causing difficulty with seeing/vision, and the length of time they had a vision problem.

### Sleep duration

Sleep duration was assessed by asking participants the following question: "On average, how many hours of sleep do you get in a 24-hour period?" Participants estimated habitual sleep duration using full hour units, i.e., 5 hours, 6 hours, and 7 hours. Sleep duration was categorized into 3 groups, short sleep duration (<6 hours), long sleep duration (>8 hours) with 6 to 8 hours of reported sleep used as the reference group [22].

### Assessment of covariates

Chronic conditions documented in the interview included hypertension, heart disease and diabetes. Body mass index (BMI) was obtained by self-reported weight and height, and categorized based on established cut-offs (i.e., obesity consistent with an index ≥ 30 kg/m^2^). Depressed mood was gathered by reporting "feeling sadness, hopelessness, worthlessness" in the previous 30 days before the face to face interviews [19].

### Statistical analysis

Statistical analysis was carried out using the Statistical Package for the Social Sciences (SPSS) (version 18.0, SPSS Inc., Chicago, IL, USA) for windows. Chi-Square test was used to compare categorical data, while ANOVA was used to compare means for continuous variables. We performed logistic regression to evaluate the associations between visual impairment and short sleep and long sleep durations relative to the reference group. In our models, we adjusted effects of age, sex, race-ethnicity, income, BMI, as well as the presence of depressed moods, hypertension, diabetes and heart disease.

## Results

Our sample consisted of 29,815 volunteers aged 18–85 years. Table 1 shows descriptive information on demographics, medical factors, sleep duration and visual impairment. The mean age was 48 years, with 56% women, and more than 70% had a family income of less than $35,000. Short sleep and long sleep durations were reported by 49% and 23% of the sample, respectively. Visual impairment was observed in 10%.

**Table 1:** **Demographic, medical factors, sleep duration and visual impairment in the National Health Interview Survey**

(%)	Total N = 29815	Black n = 4407	White N = 25408	***P***
Age, years	48 ± 18	46 ± 17	48 ± 18	<0.001
Female sex	56%	61%	55%	<0.001
Income, <35,000	77%	84%	76%	<0.001
BMI (mean ± SD)	27 ± 6	29 ± 7	27 ± 6	<0.001
Diabetes	8%	12%	8%	<0.001
Hypertension	28%	36%	27%	<0.001
Heart disease	8%	6%	9%	<0.001
Depressive symptoms	26%	29%	26%	<0.001
Visual impairment	10%	11%	10%	<0.01
Short sleep	49%	59%	48%	<0.001
Long sleep	23%	31%	22%	<0.001

The P-value indicate differences between Black and White race-ethnic groups.

Participants of White race-ethnicity constituted the majority of the sample. When compared to Whites, Blacks were younger, with a higher percentage of less than $35,000 of family income. Blacks also had an elevated BMI, as well as an increased frequency of hypertension, diabetes mellitus, depressive symptoms, visual impairment and short and long sleep durations.

Evaluation of the covariates across the sleep duration groups showed that those with either short or long sleep duration had lower incomes and a greater frequency of hypertension, diabetes mellitus, and heart disease, when compared to participants with 6–8 hours of sleep (Table 2).

**Table 2:** **Demographic, medical factors and visual impairment across sleep duration categories: National Health Interview Survey 2009**

N = 29815	< 6 h	6-8 h	> 8 h
	n = 8,454	n = 8,734	N = 2,593
	**Demographic**		
Female, n (%)	4787 (57)	4737 (54)	1539 (59)
Income, < 35,000, n (%)*	6377 (75)	6228 (71)	2261 (87)
	**Medical factors**		
Underweight, n (%)*	2821(33)	3381 (39)	983 (38)
Normal weight, n (%)*	2832 (33)	3132 (36)	869 (34)
Obesity, n (%)	2801 (33)	2221 (25)	741 (27)
Diabetes, n (%)*	768 (9)	540 (6)	332 (13)
Hypertension, n (%)	2641 (31)	2090 (24)	938 (36)
Heart Disease, n (%)*	757 (9)	617 (7)	325 (13)

*p < 0.001.

Sleep duration was associated with VI, in fully adjusted logistic regression models. Short sleep durations were associated with VI with stronger associations for long sleep in the unadjusted model. These associations were resilient to covariate adjustment: age, sex, income, and race-ethnicity, BMI, hypertension, diabetes, heart disease and depressive symptoms (Table 3). In stratified analysis, short sleep duration was associated to VI in Whites with a stronger association in Blacks. The association between long sleep and VI was mildly attenuated in Blacks when adjusting for main covariates (Table 4).

**Table 3:** **The Association between visual impairment and sleep duration in the National Health Interview Survey, 2009**

	Visual impairment	
Short sleep,* < 6 hours	OR	95% C.I
Model A	1.8	1.6-2.0
Model B	1.8	1.6-2.1
Model C	1.6	1.5-1.9
Long sleep,* > 8 hours		
Model A	2.3	2.0-2.8
Model B	1.8	1.5-2.1
Model C	1.6	1.3-1.9

*6-8 hours of sleep was the reference group.

Model A: Unadjusted.

Model B: Adjusted for age, sex, income, race-ethnicity.

Model C: Adjusted for Model B and BMI, hypertension, diabetes, heart disease, depression.

**Table 4:** **The association between visual impairment and sleep duration stratified by race-ethnic groups**

Visual impairment	White		Black	
OR (95% C.I.)	< 6 hours	> 8 hours	< 6 hours	> 8 hours
**Model A**	1.9 (1.6-2.2)	2.4 (2.0-2.8)	2.2 (1.6-3.2)	2.2 (1.4-3.4)
**Model B**	1.8 (1.6-2.1)	1.8 (1.5-2.1)	2.2 (1.5-3.1)	1.8 (1.1-2.1)
**Model C**	1.6 (1.4-1.9)	1.6 (1.3-1.9)	1.9 (1.3-2.8)	1.6 (1.0-2.5)

6-8 hours was the reference group.

Model A: Unadjusted.

Model B: Adjusted for age, sex, income.

Model C: Adjusted for Model B and BMI, hypertension, diabetes, heart disease, depression.

## Discussion

We sought to ascertain associations between sleep duration and visual impairment (VI) in a representative sample of the US adult population. Participants who reported either short or long sleep durations had increased visual impairment. To our knowledge, this is the first study to document a relation between visual impairment and extremes of sleep duration, as conceived in the present analysis.

Consistent with prior studies, visual impairment had a U-shaped association with sleep duration [6, 9, 18]. In the present study, we observed associations between both short and long sleep durations and visual impairment after adjusting for depressed moods, low socioeconomic status and factors associated with sleep apnea (a known confounder for sleep duration) such as obesity, hypertension, diabetes mellitus, and heart disease. Our findings suggest that visual impairment could be a predictor of both short and long sleep durations. Even though causality cannot be determined, proposed mechanisms by which visual impairment could be associated with short and long sleep durations include shortened photoperiod and decreased circadian entrainment among participants with visual impairments [23].

Poor circadian entrainment and decreased exposure to daylight could lead to sleep fragmentation and early awakenings causing short sleep duration. In addition, poor circadian entrainment could lead to long sleep duration and or increased time in bed by altering the timing of sleep and rest-activity patterns [4, 7, 8]. In addition, long sleep might be a compensatory mechanism among those with fragmented sleep [24]. Poor sleep quality, increased sleep disturbances, and sleep apnea, a condition that leads to sleep fragmentation, are common in patients with visual impairment (i.e., glaucoma) [25–27]. The interplay among sleep duration, visual impairment and depression is also worth considering. In a study conducted among older volunteers [28] those with decreased ambient light perception, mainly explained by decreased visual acuity, had increased mood disturbances after accounting for sociodemographic, medical factors and sleep duration. Although we did not obtain measures of visual acuity in our sample, it is plausible that decreased ambient light perception may adversely affect mood, potentially leading to extremes of sleep duration. The present study represents the first attempt to use a large, multi-ethnic representative sample of the U.S. population to assess the associations among sociodemographic variables, sleep duration and visual impairment.

However, the study has some limitations. One such limitation relates to the use of cross-sectional data, preventing inferences about causal relationships. Reverse causation is a possibility, provided that abnormal sleep habits/patterns are associated with diabetes, and cardiovascular disease, both of which are risk factors for ocular disease. It is plausible that abnormal sleep patterns may increase the risk of visual impairment by affecting systemic and ocular blood supplies, but additional research studies are necessary. We did not obtain information on the visual acuity, physical activity, and medications that could affect sleep duration in our sample. Objective measures of sleep were not obtained (i.e. actigraphy and or polysomnography). In addition, measures of sleep quality (i.e. Pittsburgh Sleep Quality Index) were not collected in our sample. Self-reported sleep duration does not allow differentiating between total sleep time (TST) and time in bed (TIB). However, TIB and objective measures of TST are highly correlated, and studies that have discriminated between both measures have found similar associations with increased mortality [29, 30].

## Conclusion

Visual impairment was associated with inadequate (short and long) sleep duration. Although prospective studies are needed to replicate the present findings, our study suggests that visual impairment should be considered as an important factor likely to influence interpretation of epidemiologic sleep data.
